# An Alternative Culture Medium for Continuous In Vitro Propagation of the Human Pathogen *Babesia duncani* in Human Erythrocytes

**DOI:** 10.3390/pathogens11050599

**Published:** 2022-05-20

**Authors:** Pallavi Singh, Anasuya C. Pal, Choukri Ben Mamoun

**Affiliations:** Department of Internal Medicine, Section of Infectious Diseases, Yale School of Medicine, New Haven, CT 06519, USA; pallavi.singh@yale.edu (P.S.); anasuya.chattopadhyay@yale.edu (A.C.P.)

**Keywords:** parasite, babesiosis, *Babesia duncani*, in vitro culture, erythrocytes, DMEM-F12, virulence

## Abstract

Continuous propagation of *Babesia duncani* in vitro in human erythrocytes and the availability of a mouse model of *B. duncani* lethal infection make this parasite an ideal model to study *Babesia* biology and pathogenesis. Two culture media, HL-1 and Claycomb, with proprietary formulations are the only culture media known to support the parasite growth in human erythrocytes; however, the HL-1 medium has been discontinued and the Claycomb medium is often unavailable leading to major interruptions in the study of this pathogen. To identify alternative media conditions, we evaluated the growth of *B. duncani* in various culture media with well-defined compositions. We report that the DMEM-F12 culture medium supports the continuous growth of the parasite in human erythrocytes to levels equal to those achieved in the HL-1 and Claycomb media. We generated new clones of *B. duncani* from the parental WA-1 clinical isolate after three consecutive subcloning events in this medium. All clones showed a multiplication rate in vitro similar to that of the WA-1 parental isolate and cause fatal infection in C3H/HeJ mice. The culture medium, which can be readily reconstituted from its individual components, and the tools and resources developed here will facilitate the study of *B. duncani.*

## 1. Introduction

The rapid emergence of tick-borne disease cases in the United States as well as other parts of the world poses a great threat to human health, thereby highlighting the need to develop novel disease diagnosis tools and novel effective therapies to treat the disease as well as strategic plans to control pathogen transmission [1]. One such disease is human babesiosis, which is caused by intraerythrocytic apicomplexan parasites of the genus *Babesia*. Human babesiosis infections are often asymptomatic or display mild flu-like symptoms in healthy individuals [2,3,4]. However, the disease can become severe and fatal in immunocompromised, asplenic and elderly individuals, with symptoms ranging from acute respiratory distress, hemolytic anemia, multiple organ failure and possibly death [2,3,4].

Several *Babesia* species have been associated with infection in humans. They include *Babesia microti* [5], *Babesia duncani* [4,6,7], *Babesia divergens* [8], *Babesia divergens* MO1 [9], *Babesia crassa*-like [10], *Babesia venatorum* [11] and *Babesia odocoilei* [12]. *B. microti* is responsible for the majority of reported clinical babesiosis cases whereas WA-1 and WA-1-type *B. duncani* cases have so far been documented primarily in Washington state and California [3,4,6].

*Babesia* parasites are transmitted to humans primarily through a bite from an infected tick or through blood transfusion [3], but transplacental transmission from mother to child can also occur [3,13].

*Babesia* spp. are phylogenetically related to *Plasmodium* spp., the causal agents of human malaria [14,15]. Both *Babesia* and *Plasmodium* spp. are obligate intracellular parasites with a complex life cycle which involves an invertebrate vector (ticks or mosquitoes) and a vertebrate host (humans). The intraerythrocytic life of *Babesia* parasites starts following invasion of red blood cells (RBCs) by free circulating merozoites. Following cell invasion, each merozoite develops and multiplies to form four daughter parasites, which subsequently exit the infected RBC (iRBC) through the process of egress. The egressed merozoites continue to infect new RBCs and increase their numbers exponentially. The repeated rounds of invasion and egress into and from the human RBCs (hRBCs) are responsible for the clinical manifestations of human babesiosis.

Efforts to control the disease through the development of novel therapeutics heavily rely on understanding the biological processes that control the development of the parasites within the host RBCs. A recent study has demonstrated the successful establishment of an in vitro culture system for the propagation of *B. duncani* in hRBCs [16]. This continuous in vitro culture system made it possible for the first time to conduct large-scale screening of chemical libraries to assess the efficacy of novel drugs, as well as to study drug–drug interactions [17]. In addition to its ability to propagate in human erythrocytes in vitro, *B. duncani* can also infect mice and causes lethal infection in both immunocompetent and immunocompromised animals. The severity of the disease and survival outcome in mice depends heavily on the genetic background and the infectious dose [18]. A combined *B. duncani* continuous in vitro culture in hRBCs and in vivo lethal infection in mice is referred to as the in culture-in mouse (ICIM) model of duncani infection and represents an ideal system to study and address several critical questions related to intraerythrocytic parasitism, host-parasite interactions, parasite virulence and disease pathogenesis [18]. The ICIM model provides a strong foundation to develop novel therapeutic strategies against babesiosis as well as diseases caused by other apicomplexan parasites. 

The success of establishment of an in vitro culture system of apicomplexan parasites depends on the nutritional requirements of the parasite. While some parasites can be propagated in basic cell culture medium such as RPMI (*Plasmodium falciparum*) or DMEM (*Toxoplasma gondii*), others require special medium such as LIT (*Trypanosoma cruzi*) [19] and NNN (*Leishmania*) [20]. Since its inception, the *B. duncani* continuous in vitro culture system in hRBCs relied on two commercially manufactured media, HL-1 (Lonza) and Claycomb (Sigma) [21]. The high cost of these complex media and the frequent shortages, which were further exacerbated by the COVID-19 pandemic, have significantly impacted the advancement of *Babesia* research. Furthermore, the composition of the HL-1 medium is unknown, and the culture medium has been unavailable since March 2021 and has now been discontinued by the manufacturer as of March 2022. Whereas the composition of the Claycomb medium was previously reported [21], the specific source of the base medium is not known and several protein components, such as growth factors, used as supplements in the medium are expensive. To overcome all these challenges, this study was conducted to identify an alternate growth medium that can support the in vitro growth of *B. duncani* in human red blood cells. Here we report that DMEM-F12 medium supports continuous in vitro culture of *B. duncani* in human red blood cells to levels identical to those achieved with HL-1 and Claycomb media. The lower cost of this culture medium and the availability of its constituent formulation, which makes it possible to make it entirely from individual components in any research lab, now eliminates completely the challenges caused by media shortages or discontinuations.

## 2. Results

### 2.1. DMEM-F12 Medium Supports the In Vitro growth of B. duncani WA-1 in Human RBCs

**Figure 1:**
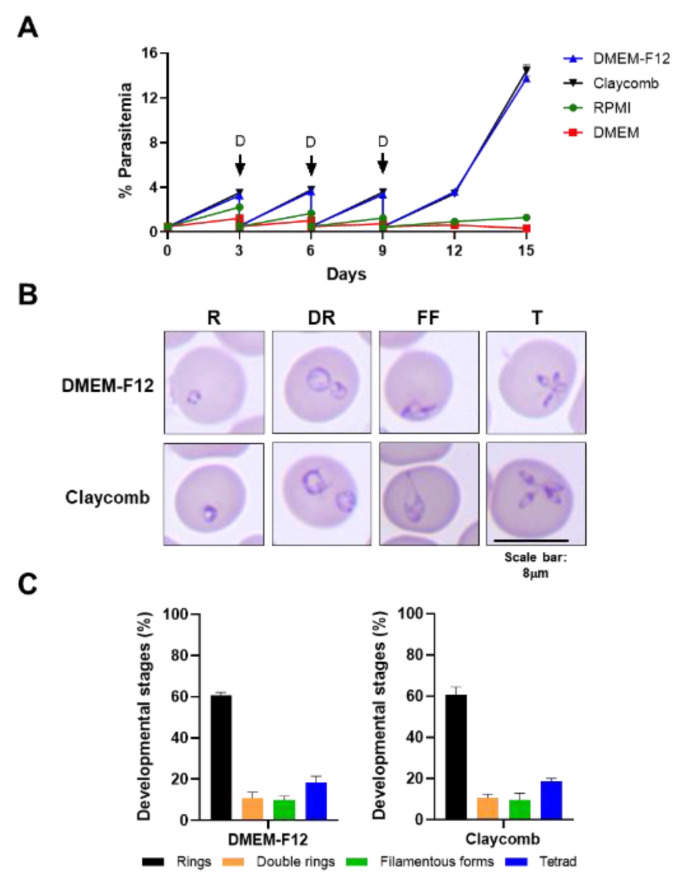
In vitro culture of *B. duncani* WA-1 in different growth media. (**A**) Growth of *B. duncani* WA-1 over a 15-day period in human RBCs with culture dilution at day 3, day 6 and day 9. Arrows (**D**) indicate when cultures were diluted to 0.5% parasitemia as determined by counting of Giemsa-stained blood smears. A total of 2500–3500 RBCs were counted. (**B**) Representative images of Giemsa-stained smears of *B. duncani* WA-1 infected human erythrocytes on day 15 in DMEM-F12 or Claycomb media showing different infection forms. R, rings; DR, double rings; FF, filamentous forms, T, tetrads. (**C**) Graph represents percentages of different parasite development stages as identified in DMEM-F12 or Claycomb media. Data presented as mean ± SD of two independent experiments performed in biological duplicates. No significant differences (*p* ≥ 0.99, two-way ANOVA) were observed between the different developmental stages in the two media.

To identify defined nutritional conditions that could support continuous in vitro propagation of *B. duncani* WA-1 clinical isolate in human RBCs, several growth media with known formulations were tested as base media, including RPMI, DMEM and DMEM-F12 from various sources, and parasite growth was compared to that under Claycomb medium. All base media were supplemented as indicated in Table 1 to build the complete culture media. The rate of parasite multiplication was determined by initiating parasite cultures in different growth media at 0.5% parasitemia in A^+^ human RBCs (5% hematocrit (HC) (day 0)) using a preculture of *B. duncani* propagated in Claycomb-based medium and washed extensively in each of the four complete media. The parasitemia was monitored by light microscopy every third day (day 3, day 6, day 9, day 12 and day 15) and the cultures were diluted to 0.5% parasitemia on days 3, 6 and 9. Monitoring of parasite counts by light microscopy showed that *B. duncani* parasitemia increased to similar levels in Claycomb- and DMEM-F12-based media on days 3, 6, 9, 12 and 15 (Figure 1A). However, in both RPMI- or DMEM-based complete media, *B. duncani* parasitemia showed modest increase during the first cycle of continuous growth ending on day 3 post-inoculation but no significant increase was detected during the following cycles ending on day 6, day 9, day 12 and day 15 (Figure 1A). Parasite morphology was identical in both DMEM-F12- and Claycomb-based media with all developmental stages represented under both growth conditions (Figure 1B). The proportion of infected red blood cells with rings, double rings, filamentous forms and tetrads throughout the intraerythrocytic cycle was comparable in both media with no significant differences (*p* ≥ 0.99, two-way ANOVA) observed between the different developmental stages in the two media (Figure 1C).

### 2.2. Derivation of New Lines of B. duncani WA-1 Clinical Isolate

**Figure 2:**
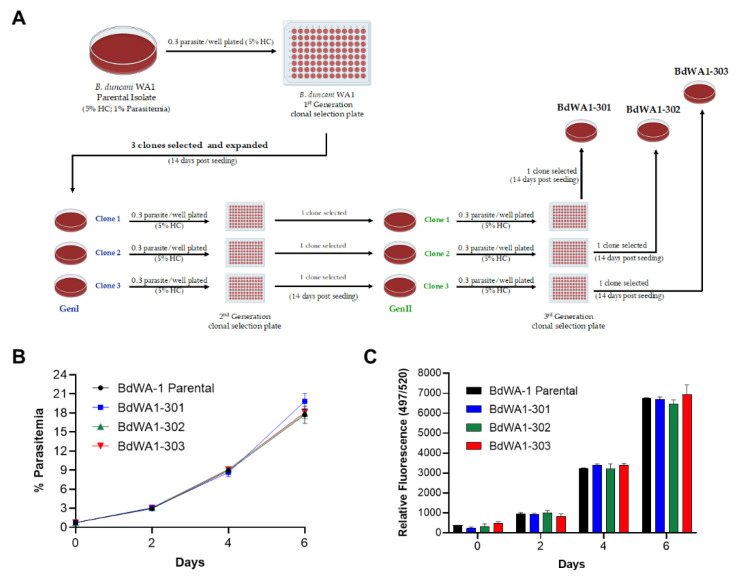
Limiting dilution cloning of *B. duncani* parental isolate and growth comparison of triple cloned and in vitro cultured *B. duncani* WA-1 clones to parental isolate. (**A**) Schematic representation of limiting dilution cloning of *B. duncani* WA-1 parasites. The parental clinical *B. duncani* WA-1 parasite was subcloned by three consecutive limiting dilution steps to produce first, second and third generation clones. (**B**,**C**) Multiplication rate of BdWA1-301, BdWA-2 and BdWA-3 clones in human RBCs in DMEM-F12-based complete culture medium. The multiplication rate of the third generation clones was compared to that of the parental BdWA-1 strain and determined by microscopic examination of Giemsa-stained smears from samples collected at the indicated time points (**B**) and using the SYBR Green I incorporation assay (**C**). The data represent mean ± SD of biological duplicates. No significant differences were found in the parasitemia counts by microscopy (*p* ≥ 0.1, two-way ANOVA) or by SYBR Green I incorporation (*p* ≥ 0.6, two-way ANOVA) between *B. duncani* parental isolate and three GenIII clones.

**Figure 3:**
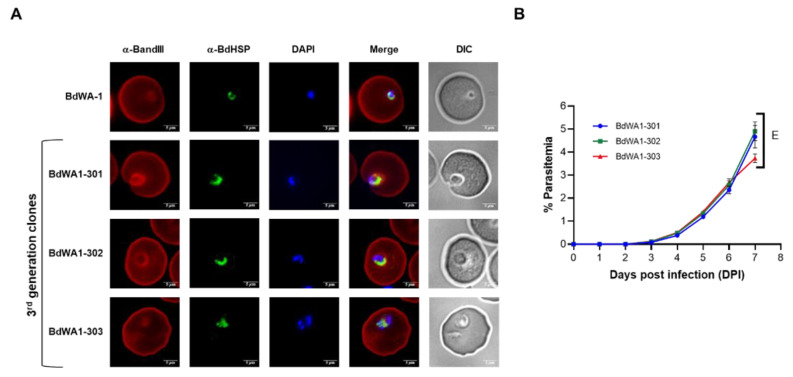
Cloned *B. duncani* WA-1 parasites show similar expression profile of *B. duncani* protein and maintain virulence in in vivo mouse model. (**A**) Subcellular localization of a putative BdHSP70 (ID#: BdWA1_001707) protein of *B. duncani* in the parental isolate and third generation clones. Images show immunofluorescence staining of BdHSP70 protein with polyclonal antibodies raised against the protein in rabbits followed by Alexa Fluor 488 conjugated anti-rabbit immunoglobulin secondary antibodies on fixed human RBCs infected with either BdWA-1 or third generation clones BdWA1-301, BdWA1-302 and BdWA1-303. DAPI was used to label parasite DNA. Human RBCs were stained with an anti-BandIII antibody followed by Rhodamine conjugated anti-human secondary antibody. Bars, 5 μm. (**B**) Evaluation of the virulence of third generation BdWA1-301, BdWA-2 and BdWA-3 clones in C3H/HeJ mice (three mice per clone) following intravenous administration of 10^7^ infected human RBCs. The multiplication rate of the third generation clones was determined by microscopic examination of Giemsa-stained smears of mouse blood collected at the indicated time points and no significant differences between the parasitemia levels were observed (*p* > 0.2, two-way ANOVA) between the different infected groups. E, euthanized.

The *B. duncani* WA-1 clinical isolate was cryopreserved from blood collected in 1991 from a patient from Washington state [22]. The sample was found to be highly virulent following inoculation into hamsters and caused acute infection and death in animals [23]. WA-1 was subsequently used to inoculate both hamsters and mice to study the immune response to *B. duncani* as well as to grow the parasite in vitro in human or hamster red blood cells. To ensure the clonality of the parasite, we conducted three consecutive limiting dilution cloning of the WA-1 isolate in the DMEM-F12-based complete culture medium as shown in Figure 2. Each dilution cloning was conducted in a 96-well plate format with 0.3 infected red blood cell per well at 5% HC. Culture medium was changed every third day and the parasitemia was monitored after 14 days by light microscopy of Giemsa-stained blood smears. Six individual clones were isolated in the first round of cloning, three of which were subjected to the second round of limiting dilution cloning. Three individual clones were isolated in the second round of cloning and were subjected to a third round of limiting dilution cloning. The last three clones isolated are referred to as BdWA1-301, BdWA1-302 and BdWA1-303. Comparison of growth kinetics in DMEM-F12-based medium showed that all clones have similar growth patterns in vitro in hRBCs compared to the BdWA-1 parental isolate as determined by parasitemia count determined by light microscopy (Figure 2B) or by increased incorporation of SYBR Green in parasite DNA (Figure 2C). No significant differences were found in the parasitemia counts by microscopy (*p* ≥ 0.1, two-way ANOVA) or by SYBR Green I incorporation (*p* ≥ 0.6, two-way ANOVA) between *B. duncani* parental isolate and three GenIII clones. Additionally, an immunofluorescence assay performed to localize a predicted heat shock protein, BdHSP70 (ID#: BdWA1_001707), using affinity-purified antisera showed that the localization of this protein in all three clones follows the same pattern as the parental clone, indicating that there are no major differences in the protein expression between these clonal lines (Figure 3A).

### 2.3. New B. duncani Clones Are Virulent in Mice

Our recent work on the *B. duncani* ICIM model demonstrated the infectivity of in vitro cultured parental BdWA-1 clinical isolate when inoculated into mice [18]. To assess whether the newly cloned BdWA1-301, BdWA1-302 and BdWA1-303 parasites are still virulent in mice, the parasites were propagated in vitro in human red blood cells and injected by the intravenous (IV) route into female C3H/HeJ mice (*n* = 3/clone). Parasitemia was monitored by determining parasite counts in Giemsa-stained thin blood smears made from blood collected from infected mice. As shown in Figure 3B, all three clones led to establishment of parasitemia in mice starting at DPI3 demonstrating the infectivity of all third generation *B. duncani* clones. Peak parasitemia in all mice ranged between 3.6% and 5.2% at DPI 7 at which point all mice became moribund and were euthanized. No significant differences between the parasitemia levels were observed (*p* > 0.2, two-way ANOVA) between the different infected groups.

## 3. Discussion

In this short communication, we report the identification of DMEM-F12-based culture medium as an alternative growth medium for continuous propagation of *B. duncani* in human red blood cells. No parasite adaptation was required when shifting the parasite from Claycomb or HL1-based media to DMEM-F12. Furthermore, we have now successfully propagated *B. duncani* in continuous in vitro culture in human red blood cells in this medium for several months. The discovery of the DMEM-F12 medium will help the *Babesia* research community overcome the major challenges faced over the past several years as a result of frequent shortages by the manufacturers of the HL-1 and Claycomb media. These two media were first reported to be suitable for propagation of the parasite in human and hamster red blood cells in vitro [16,24]. However, their specific constituents were not disclosed by the manufacturers. While media shortages were common before the COVID-19 pandemic, the situation worsened during the pandemic with backorders for both media announced for several months. To add “insult to injury”, one of the manufacturers announced discontinuation of the HL-1 medium. Faced with these challenges, we scrambled to reconstitute the Claycomb medium from the constituents reported in the original report by Claycomb and colleagues for propagation of human HL-1 cardiomyocytes [21,25]. This growth condition uses DMEM as a base medium supplemented with proteins and lipids; however, the specific DMEM used was not indicated [21]. When reconstituted based on reported formulation and using different commercially available DMEM media, no growth of *B. duncani* could be achieved in culture. Interestingly, we found that DMEM-F12-based culture medium, without the supplements used in the Claycomb media, was sufficient to support the continuous growth of *B. duncani* in hRBCs. This finding suggests that *B. duncani* relies on specific nutrients from its environment for survival within human red blood cells and some of these nutrients are present in the DMEM-F12 but not the standard DMEM medium. Analysis of the components of these two media identified several nutrients that are present in DMEM-F12 but absent in DMEM. These include six amino acids (alanine, asparagine, aspartic acid, cysteine, glutamic acid and proline), two vitamins (biotin and cobalamin) and four inorganic salts (cupric sulfate, ferric sulfate, magnesium chloride and zinc sulfate) as well as hypoxanthine, thymidine, linoleic acid, lipoic acid and putrescine. A new study is now underway to determine which of these nutrients is critical for *B. duncani* survival in human red blood cells. The reliance of *B. duncani* on host nutrients for survival is shared with other intraerythrocytic parasites such as the human malaria parasite *P. falciparum* [26,27]. The malaria parasite relies heavily on a large number of nutrients such as carbohydrates, lipid precursors and vitamins for survival within human erythrocytes. The transporters and metabolic enzymes involved in the uptake and utilization of these nutrients are considered attractive targets for the development of new antimalarial drugs [26,28,29,30,31]. Future efforts to identify the nutrients required for *Babesia* survival in human erythrocytes and to understand their mechanism of uptake and utilization are warranted.

The discovery that DMEM-F12 supports the continuous growth of *B. duncani* in vitro is also important for two other reasons. First, DMEM-F12 is more affordable than the HL-1 and Claycomb media. Second, the availability of a detailed composition of the DMEM-F12 medium makes it possible to reconstitute it entirely from individual constituents in any research lab thus preventing any possible disruptions that could occur in the future due to manufacturers’ shortages or discontinuations.

Finally, we report the isolation of three third generation clones of *B. duncani* from the WA-1 clinical isolate. These clones represent an important resource for the scientific community as more scientists become interested in the biology, pathogenesis and virulence of *Babesia* parasites. The ability of *B. duncani* to grow continuously in vitro in human red blood cells and to cause severe disease and fatal infection in mice (ICIM model [18]) is unique among apicomplexan parasites that infect human red blood cells and creates an unprecedented opportunity to evaluate the efficacy of experimental drugs in vitro and in mice to accelerate research towards the development of effective antiparasitic therapies [3,17,18].

The tools and resources reported in this communication are available to the research community through direct requests and will be shared with BEI Resources for wider distribution.

## 4. Materials and Methods

### 4.1. In Vitro Parasite Culture of B. duncani WA-1 in Different Growth Media

*B. duncani* parasites were cultured in vitro as reported previously [16]. Parasite growth was monitored and evaluated in the following media: DMEM (Thermo Fisher, 11995-065, Waltham, MA, USA), DMEM-F12 (Lonza, BE04-687/U1, Basel, Switzerland), RPMI (Thermo Fisher, 11875-093) and Claycomb (Sigma, 51800C, Grand Island, NY, USA). All media were supplemented with 20% heat-inactivated FBS (Gibco, 10438-026, Waltham, MA, USA), 1× of 50× HT Media Supplement Hybrid-Max ^TM^ (Sigma, H0137), 1× of 100× (200 mM) L-Glutamine (Gibco, 25030-081), 1× of 100× Antibiotic-Antimycotic (Gibco, 15240-062) and 1× of 100× (10 mg/mL) Gentamicin (Gibco, 15710-072).

### 4.2. In Vitro Culture of B. duncani in Human RBCs

In vitro propagation of *B. duncani* in hRBCs was carried out as previously reported by Abraham et al. [16] and Chiu et al. [17]. Parasitemia was monitored either by light microscopy examination of Giemsa-stained blood smears or by fluorescence detection of SYBR Green I [16].

### 4.3. Cloning of B. duncani WA-1 Clinical Isolate

In vitro culture of *B. duncani* WA-1 clinical isolate was initiated in A^+^ human RBCs in DMEM-F12 medium at 0.5% parasitemia and 5% hematocrit (HC). The parasites were allowed to grow for 2 days and the parasitemia was measured by Giemsa-stained blood smears on day 2. The culture was serially diluted to obtain 30 parasites in 20 mL volume at 5% HC. Then, 200 μL of this parasite suspension was plated per well of a 96-well plate. This GenI cloning plate was subjected to medium change on every third day. To determine which wells of the 96-well plate contain parasites, the parasitemia estimation was performed using SYBR Green-I based fluorescence assay on day 14. Then, 25 μL of culture per well of the 96-well plate was mixed with 25 μL of SYBR Green-I lysis buffer consisting of 20 mM Tris, pH 7.4, 5 mM EDTA, 0.008% saponin, 0.08% Triton X-100 and 1X SYBR Green-I (Molecular Probes, 10,000X solution in DMSO, Eugene, OR, USA). Additionally, the uninfected human RBCs (5% HC, 25 μL volume) were used as a negative control. The SYBR Green-I measurement plates were incubated in dark at 37 °C for 1 h. Following this, the plates were read on a BioTek Synergy MX fluorescence plate reader with an excitation of 497 nm and emission of 520 nm. Using readings from uninfected human RBC as a background, the readings for different wells of GenI cloning plate were calculated to determine which wells of the 96-well plate contained parasites (higher SYBR Green-I readings in comparison to the negative control). After identification of the wells containing parasite clones, Giemsa smears were prepared from the same wells and observed under light microscopy to confirm the presence of the parasites. Following this, six GenI *B. duncani* clones were picked and expanded to 1 mL cultures. The clones were allowed to grow to a parasitemia of 2%. Three out of six clones were chosen to perform a second round of limiting dilution cloning using the same steps as described above for GenI cloning. On the 14th day, the GenII clonal plates were screened for parasites using SYBR Green-I based assay. Three Gen II clones were picked, expanded to 1 mL cultures and allowed to grow to 2% parasitemia. Finally, these three GenII clones were subjected to a final third round of limiting dilution cloning and three GenIII clones were obtained after day 14. These three GenIII clones were named as BdWA1-301, BdWA1-302 and BdWA1-303.

### 4.4. Comparison of In Vitro growth of B. duncani WA-1 and Clonal Lines BdWA1-301, BdWA1-302 and BdWA1-303

In vitro cultures of BdWA-1, BdWA1-301, BdWA1-302 and BdWA1-303 parasites were initiated in A^+^ human RBCs in DMEM-F12 medium at 0.75% parasitemia and 5% HC. The parasites were allowed to grow for 6 days, and the media was changed after every 24 h. The parasitemia was measured by SYBR Green I based assay as well as counting of Giemsa-stained blood smears on day 2, day 4 and day 6. Data were analyzed using Graphpad Prism version 9.3.1 software. Error bar represents mean ± SD of two independent experiments performed in biological duplicates.

### 4.5. Immunofluorescence Assay

Thin blood smears from in vitro cultures of BdWA1-301, BdWA1-302, BdWA1-303 and Bd WA-1 parental isolate were prepared on glass slides (640-001T, DOT Scientific, Tokyo, Japan) and fixed with chilled methanol (9070-05, JT Baker) for 15 min at −20 °C. The smears were air-dried and blocked in 3% BSA in PBS buffer (A9418, Sigma) for 1 h at room temperature. Following this, the smears were incubated with rabbit polyclonal anti-HSP70 (BdWA1_001707) antibodies (1:200 dilution) and mouse monoclonal anti-Band3 antibody (1:500) (Sigma, B9277) for 1 h at room temperature. This was followed by three washes in 1X PBS containing 0.05% Tween (PBST) and three washes in 1X PBS, 5 min each. Subsequently, the smears were incubated with goat anti-rabbit IgG antibodies conjugated to Alexa Fluor 488 (1:500 dilution) (A-11008, Life Technologies) and goat anti-mouse IgG (H+L) antibodies conjugated to Rhodamine (1:500 dilution) (31660, Invitrogen, Waltham, MA, USA) for 1 h at room temperature. This was followed by three washes in 1X PBST and three washes in 1X PBS. Coverslips were then mounted on the slides using Vectashield mounting medium containing DAPI (H-1200-10, Vector Laboratories, Burlingame, CA, USA) and observed under Nikon ECLIPSE TE2000-E microscope. A 100X oil immersion objective was used for image acquisition. Excitation at 465–495 nm was used to detect Alexa Fluor 488 positive cells; excitation at 510–560 nm was used to detect Rhodamine positive cells and excitation at 340–380 nm was used to detect DAPI positive cells. The images were acquired using MetaVue with 1392 × 1040 pixel as the chosen image size and subsequently analyzed using ImageJ.

### 4.6. Ethics Statement

All animal experiments were approved by the Institutional Animal Care and Use Committees (IACUC) at Yale University (Protocol #2020-07689). Animals were acclimatized for 1 week after arrival before the start of an experiment. Animals that showed signs of distress or appeared moribund were humanly euthanized using approved protocols.

### 4.7. Virulence Assays in Mice

To assess the virulence of third generation *B. duncani* clones, 6-to-8-week-old female C3H/HeJ mice (The Jackson Laboratories, Bar Harbor, ME, USA) were inoculated with 1 × 10^7^ iRBC of BdWA1-301, BdWA1-302 or BdWA1-303 (*n* = 3/clone) by the IV route. Parasitemia was monitored over time by light microscopic examination of Giemsa-stained blood smears. Moribund mice were humanely euthanized.

## Figures and Tables

**Table 1:** Constituents of the culture media that support continuous growth of *B. duncani* in human RBCs in vitro.

	Complete Claycomb Medium	Complete DMEM-F12 Medium	Complete HL-1 Medium
**Base Medium**	Claycomb Medium (Sigma: Cat. No.: 51800C)	DMEM-F12 Medium (Lonza: Cal. No.: BE04-687F/U1)	HL-1 Medium (LonzaTM: Discontinued)
**Supplements**	20% Fetal Bovine Serum (Heat Inactivated) (Gibco, Cat. No.: 10438-026)		
	1× of HT media supplement (50×) (Sigma, Cat. No.: H0137-10VL)		
	1× of L-Glutamine (200 mM; 100×) (Gibco, Cat. No.: 25030-081)		
	1× of Antimycotic-Antibiotic (100×) (Gibco, Cat. No.: 15240-062)		
	1× of Gentamicin (10 mg/mL; 100×) (Gibco, Cat. No.: 15710-072)		

## Data Availability

Not applicable.
